# Zika Virus Induces Sex-Dependent Metabolic Changes in *Drosophila melanogaster* to Promote Viral Replication

**DOI:** 10.3389/fimmu.2022.903860

**Published:** 2022-06-30

**Authors:** Ghada Tafesh-Edwards, Ananda Kalukin, Ioannis Eleftherianos

**Affiliations:** Infection and Innate Immunity Laboratory, Department of Biological Sciences, The George Washington University, Washington, DC, United States

**Keywords:** *Drosophila*, Zika virus, innate immunity, RNA interference, metabolism

## Abstract

Zika is a member of the *Flaviviridae* virus family that poses some of the most significant global health risks, causing neurologic complications that range from sensory neuropathy and seizures to congenital Zika syndrome (microcephaly) in infants born to mothers infected during pregnancy. The recent outbreak of Zika virus (ZIKV) and its serious health threats calls for the characterization and understanding of Zika pathogenesis, as well as host antiviral immune functions. Although ZIKV has been associated with activating the RNA interference (RNAi) immune pathway and altering host metabolism, in-depth studies are still required to uncover the specifics of the complex host-virus interactions and provide additional insights into the molecular components that determine the outcome of this disease. Previous research establishes the fruit fly *Drosophila melanogaster* as a reliable model for studying viral pathogens, as it shares significant similarities with that of vertebrate animal systems. Here, we have developed an *in vivo Drosophila* model to investigate ZIKV-mediated perturbed metabolism in correlation to the RNAi central mediator Dicer-2. We report that ZIKV infection reprograms glucose and glycogen metabolism in *Dicer-2* mutants to maintain efficient replication and successful propagation. Flies that exhibit these metabolic effects also show reduced food intake, which highlights the complicated neurological defects associated with ZIKV. We show that ZIKV infection significantly reduces insulin gene expression in *Dicer-2* mutants, suggesting an insulin antiviral role against ZIKV and a direct connection to RNAi immunity. Moreover, we find that the insulin receptor substrate c*hico* is crucial to the survival of ZIKV-infected flies. These observations are remarkably more severe in adult female flies compared to males, indicating possible sex differences in the rates of infection and susceptibility to the development of disease. Such findings not only demonstrate that metabolic alterations can be potentially exploited for developing immune therapeutic strategies but also that preventive measures for disease development may require sex-specific approaches. Therefore, further studies are urgently needed to explore the molecular factors that could be considered as targets to inhibit ZIKV manipulation of host cell metabolism in females and males.

## Introduction

With World Health Organization (WHO) scientists confirming recent outbreaks of Zika virus (ZIKV) in India, investigating and understanding the molecular mechanisms underlying immune responses against ZIKV have become ever more urgent (1, 2). Zika, a single-stranded positive-sense RNA virus and a member of the *Flaviviridae* family, is transmitted to humans primarily by the infected *Aedes* mosquito species (*Ae*. *aegypti* and *Ae*. *albopictus*) and associated with abnormal functions of neuronal cells leading to neurological disorders such as microcephaly in newborns and Guillain-Barreí syndrome in adults (3, 4). Since 2015, ZIKV spread rapidly and created an enormous global alarm, with more than 1,000,000 estimated cases occurring in the Americas (5). There are currently no licensed treatments or vaccines effective against ZIKV infection. Hence, the development of an *in vivo* model to uncover the complex host-ZIKV interactions and identify the molecular components involved in immune signaling are crucial to the advancement of innovative concepts and means for the efficient control of ZIKV disease and clarifying anti-ZIKV immune mechanisms in humans.

The fruit fly *Drosophila melanogaster* has proven to be a significant model organism for researching cellular interactions of various human pathogens including arboviruses (arthropod-borne viruses) such as ZIKV and investigating the genetic basis of antiviral resistance (6–10). Particularly, the large conservation between *Drosophila* and mosquitoes has already paved the way for the recent, novel insights into Zika pathogenesis and host immune function using *Drosophila* models (11–14). With the availability of well-established techniques for developmental and genetic manipulations, *Drosophila* is considered a potent model for studying host-pathogen interactions and innate immunity, which shares significant similarities with that of vertebrate animal systems (15, 16). As in vertebrates, numerous *Drosophila* immune responses are triggered by diverse pathogen infections, leading to the transcriptional induction of downstream NF-κB-dependent effectors including antimicrobial peptides (AMPs) that can attenuate or clear infection (17, 18). These antibacterial and antifungal signaling mechanisms, regulated by the inflammatory NF-κB pathways Toll and immune deficiency (IMD), are well-characterized and documented (19, 20). However, their roles in antiviral defense remain poorly understood. Little is also known about the roles of other major humoral and cellular immune pathways such as JAK/STAT, autophagy, and melanization (21, 22).

The central antiviral immune response in the fly involves the canonical RNA interference (RNAi) pathway, a highly conserved nucleic acid–based immune defense that is induced by double stranded RNA (dsRNA) to mediate sequence-specific gene silencing (23–25). In *Drosophila*, RNAi involves the RNase Dicer-2 that recognizes and cleaves dsRNA to generate viral small interfering RNAs (siRNAs). These siRNAs are loaded onto Argonaute-2 (Ago-2) protein to guide the RNA-induced silencing complex (RISC) to complementary RNAs in the cell, leading to their sequence-specific degradation. Recent findings show that upon infection with ZIKV, flies display a substantial increase in viral replication that does not compromise their survival (11, 26). Most importantly, *Dicer-2* mutants display enhanced ZIKV load and increased susceptibility to ZIKV infection, suggesting that RNAi confers resistance to ZIKV infection in *Drosophila* (11). ZIKV is also confirmed to exhibit tissue tropism by infecting the fat body, crop, and gut of the adult fly, which results in local pathologies marked by changes in the host homeostasis and metabolism (11). These alterations indicate a significant role of metabolism in the defense against ZIKV, thereby stressing the need for a more comprehensive look into the intricacies of host–ZIKV dynamics to understand the molecular and physiological processes that determine the outcome of this disease.

In this study, we used a *Drosophila* model system to study the interplay between host immunometabolism and ZIKV infection. We show that not only does ZIKV reduce feeding in infected flies but also trigger internal changes such as carbohydrate metabolism in *Dicer-2* mutant flies. These fluctuations during infection confirm perturbed host metabolism and homeostasis, which pathogens manipulate to acquire their specific metabolic needs (27). We also find that insulin signaling, one of the main metabolic pathways involved in host-pathogen interactions, is targeted by ZIKV in *Dicer-2* mutants, therefore reporting an antiviral role for insulin during ZIKV infection and a direct correlation to the RNAi pathway. In addition, we show for the first time that the insulin receptor substrate *chico* is required for the survival of ZIKV-infected flies. Interestingly, these observations were sex-dependent, which highlights the importance of using both male and female animals for *in vivo* efficacy studies. Overall, this work identifies host carbohydrate metabolism and insulin signaling as components of *Drosophila* immunity against ZIKV infection and demonstrates that insulin works cooperatively with the RNAi antiviral immune pathway for host protection. These are important findings that give more insight into the arms race between hosts and pathogens in general and more specifically the mechanisms *Drosophila* and ZIKV use to regulate metabolic pathways to their own advantage.

## Materials and Methods

### Fly and Nematode Stocks

*Dicer-2* ^L811fsX^ and *chico* ^14337^ were compared to a *YW* background control. *dFOXO* ^C01841^ was also used in comparison to *w^1118^* background control. All flies were reared on Bloomington *Drosophila* Stock Center cornmeal food (LabExpress) supplemented with yeast (Carolina Biological Supply), and maintained at 25°C with a 12:12-h light:dark photoperiodic cycle.

### Fly Infection

ZIKV strain MR766 was prepared as previously described (11). Injections were performed by anesthetizing flies with carbon dioxide on a gas pad. For each experiment, 2–5-day-old adult males and female flies were sorted into separate vials and injected with ZIKV suspensions in PBS (pH 7.5) using a nanoinjector (Nanoject III; Drummond Scientific). Heat-inactivated or live ZIKV solution (11,000 PFU/fly) (100 nl) was injected into the thorax of flies, and an injection of the same volume of PBS acted as negative control. Injected flies were then maintained at 25°C and transferred to fresh vials every third day throughout the experiment. They were collected at 4 days post injection and directly used for other experiments in this study. Statistical analyses of the differences in gene expression between fly strains and experimental conditions were conducted with duplicate replicates from three independent experiments.

### Fly Survival

For each fly strain, three groups of 20 male and female flies were injected with ZIKV and control groups were injected with PBS. Following injection, flies were maintained at a constant temperature of 25°C with a 12-hour light/dark cycle, and mortality was recorded daily. Fly deaths occurring within 1 day of injection were attributed to injury and were not included in the results.

### Gene Expression Analysis

Total RNA was extracted from 10 male or female flies, using TRIzol according to manufacturer’s protocol. Total RNA (500 ng–1 mg) was used to synthesize cDNA using the High-Capacity cDNA Reverse Transcription Kit (Applied Biosystems). Quantitative RT-PCR (qRT-PCR) experiments were performed with two technical replicates and gene-specific primers (**Table 1**) using a CFX96 Real-Time PCR detection system (Bio-Rad Laboratories). Cycle conditions were as follows: 95°C for 2 min, 40 repetitions of 95°C for 15 s followed by 61°C for 30 s, and then one round of 95°C for 15 s, 65°C for 5 s, and finally 95°C for 5 s. Quantification was performed from three biological replicates for both test and control treatments. Fold changes were calculated with the

**Table 1:** List of the gene-specific primers used in quantitative qPCR experiments.

Gene	Accession Number	Primer	Sequence (5’ - 3’)
RpL32	CG7939	Forward Reverse	GATGACCATCCGCCCAGCA CGGACCGACAGCTGCTTGGC
foxo	CG3143	Forward Reverse	AGGCGCAGCCGATAGACGAATTTA TGCTGTTGACCAGGTTCGTGTTGA
chico	CG5686	Forward Reverse	GCGCACTCACCTTATGACCA GCACACGAATGTCAGGGATTT
dilp2	CG8167	Forward Reverse	TCCACAGTGAAGTTGGCCC AGATAATCGCGTCGACCAGG
InR	CG18402	Forward Reverse	ACCTATTTAACCACAAGCGA CTCGATAGTTCCAAGATTGC
Spargel	CG9809	Forward Reverse	GGATTCACGAATGCTAAATGTGTTCC GATGGGTAGGATGCCGCTCAG
Thor	CG8846	Forward Reverse	CATAGCAGCCACACAAGCTC GGTGAAGCGGACATCTTAGC
Dicer-2	CG6493	Forward Reverse	GTATGGCGATAGTGTGACTGCGAC GCAGCTTGTTCCGCAGCAATATAGC
Argonaute-2	CG7439	Forward Reverse	CCGGAAGTGACTGTGACAGATCG CCTCCACGCACTGCATTGCTCG
NS5	055839	Forward Reverse	CCTTGGATTCTTGAACGAGGA AGAGCTTCATTCTCCAGATCAA

$2_{\text{T}}^{-\Delta \Delta C}$ method using *Ribosomal protein L32* (*RpL32*) as a housekeeping gene (28, 29).

### Feeding Assays

Food intake measurements were performed as described in prevalent feeding protocols using dye-labeled food (30). For each experimental condition, 20 male and female flies were maintained separately on the previously mentioned food for 4 days post infection. Flies were then transferred to vials with identical media containing 1% (w/v) FD&C Blue #1 (Spectrum Chemical). After 1 hour, feeding was interrupted by freezing the vials at −80°C. Frozen flies were transferred to 1.7 mL Eppendorf tubes and homogenized with a pestle on ice in 50 μl of 1x PBS + 1% Triton X-100. After centrifugation for 15 min at 9000 × g and 4°C to clear the debris, the absorbance of the supernatant was measured at 630 nm (A630) on a NanoDrop 2000 Spectrophotometer (Thermo Fisher Scientific). Flies fed non-labeled food were used as controls and their A630 values were subtracted from experimental readings. Serial dilutions of an initial 10 μl aliquot of the non-solidified dye-labeled food added to 0.99 ml of 1x PBS + 1% Triton X-100 were used to generate a standard curve. After determining the equivalent dye concentration of each homogenate using the linear fit of the standard curve, consumption was calculated by multiplying with the homogenate volume (50 μl) and dividing by the number of flies per sample.

### Glucose, Glycogen, and Trehalose Levels

Previously established enzymatic-based methods were used to quantify basic metabolites in ZIKV-infected flies (31). At 4 days post infection, 5 flies were collected for each experimental condition and rinsed several times with 1 ml of 1x PBS. Flies were then homogenized with a pellet pestle on ice in 100 μl of 1x PBS to determine glucose and glycogen levels, or in 100 μl of Trehalase buffer (TB; 5 mM Tris pH 6.6, 137 mM NaCl, 2.7 mM KCl) to measure trehalose levels.

Glucose and glycogen levels were measured by initially diluting the samples 1:3 in PBS and then further diluting them 1:1 in either amyloglucosidase stock solution (1.5 ml of amyloglucosidase in 1 ml of PBS, Sigma-Aldrich) or PBS. Diluted samples (30 μl) were loaded in duplicate rows onto a clear 96-well plate and incubated at 37°C for 60 min. Hexokinase reagent (100 μl; Glucose Assay Reagent, Sigma-Aldrich; G3293, St. Louis, MO, USA) was added to each well and samples were incubated at room temperature for an additional 15 min. Absorbance was measured at 340 nm. Glucose standard curve was used to determine glucose levels, whereas glycogen levels were calculated by subtracting the absorbance of glucose from the absorbance of samples diluted with amyloglucosidase stock.

To determine trehalose content, samples were diluted 1:3 in TB, followed by 1:1 dilution in either TB or Trehalase Stock (TS; 3 μl of porcine trehalase in 1 ml of TB). Samples (30 μl) were incubated in duplicate rows in a clear 96-well plate at 37°C for 24 hours, after which hexokinase (100 μl) was added to each well and the absorbance was measured at 340 nm. The measured absorbance for free glucose in the untreated samples (containing TB) was first subtracted from the absorbance of the samples that were digested with trehalase (TS). Trehalose levels were then calculated based on a trehalose standard curve.

Glucose, glycogen, and trehalose measurements were normalized to the amount of proteins in each sample. Protein quantification was determined by Pierce™ BCA Protein Assay Kit (Thermo Fisher Scientific; 23227, Waltham, MA, USA). Each experiment was run in technical duplicates and repeated three times.

### Cholesterol Quantification

Cholesterol levels were determined using Amplex Red Cholesterol Assay Kit (Invitrogen; A12216, Carlsbad, CA, USA). At 4 days post infection, 10 adult male and female flies were collected separately and homogenized with a pestle on ice in 100 μl of 1x reaction buffer provided by assay kit. In a black 96-well plate (Fisher Scientific; 509051574), 50 μl of each sample were mixed with 90 μl of the reaction mix provided by the kit and incubated for 30 min at 37°C. Fluorescence was measured with excitation at 530 nm and emission at 590 nm. A standard curve was used to determine cholesterol levels, and the assay was repeated three times. All data were calculated relative to the proteins in each sample. Protein quantification was determined by PierceTM BCA Protein Assay Kit (Thermo Fisher Scientific; 23227, Waltham, MA, USA).

### Statistical Analysis

All statistical analyses were performed using GraphPad Prism 9 software. Gene expression and metabolic analyses were compared using a one-way ANOVA and Bonferroni multiple comparisons test to determine differences between specific treatments. Survival curves were assessed using a Log-Rank (Mantel-Cox) test within the GraphPad Prism program. All analyses were performed on data accumulated through three independent experiments.

## Results

### ZIKV Infection Reduces Feeding in *Drosophila* Hosts

**Figure 1:**
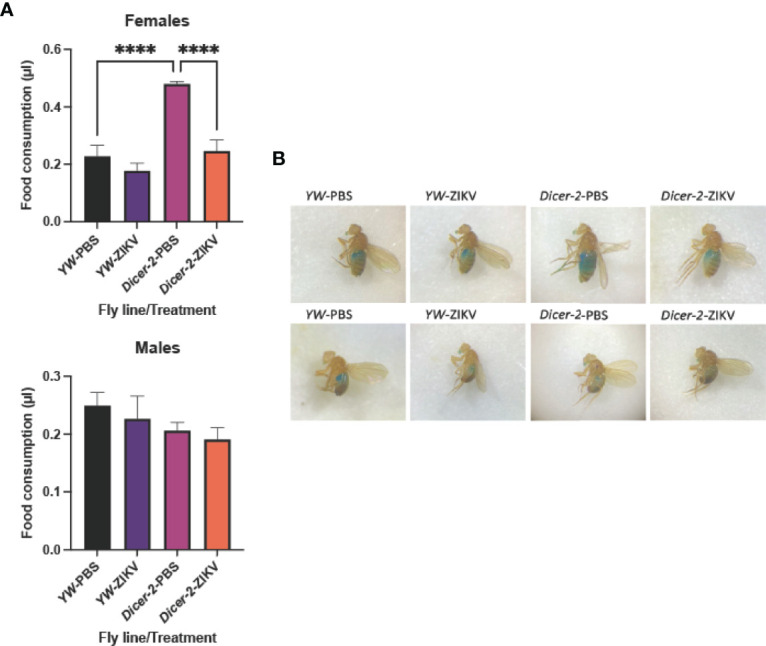
Feeding rate of *Drosophila melanogaster* upon infection with Zika virus. **(A)** *Drosophila* female and male adult *Dicer-2* mutants and their background controls (*YW*) were injected with ZIKV (African strain MR766; 110 million PFUs/ml), and feeding was estimated at 4 days post infection. Injections with PBS served as negative controls. **(B)** Dye-stained food in the abdomen of ZIKV-injected or PBS-injected *Dicer-2* mutants and *YW* individuals. Data represent three biological replicates of at least 20 flies for each experimental condition. (One-way ANOVA, ^****^p < 0.0001).

Behavioral plasticity during infections has been closely linked to host fitness and pathogen spread, thus making the integration of behavioral variation into our understanding of pathogenic infections imperative (32–34). To investigate the interplay between ZIKV infection and host behavior, we injected 11,000 PFU/fly of the strain MR766 into the thorax of *Dicer-2* mutants and their *YW* controls. We estimated food consumption, a fundamental behavioral parameter, by performing a quantitative assay that involves labeling food with FD&C Blue, a soluble, non-absorbable dye (30). Food intake measurements were taken at 4 days post injection for infected female and male adult flies. We found significantly lower consumption rates in ZIKV-infected, *Dicer-2* female mutants compared to PBS control–treated flies whereas males showed no differences in feeding trends (**Figures 1A, B**). To exclude possible effects due to PBS injections, we confirmed similar feedings rates in untreated *Dicer-2* and *YW* flies (**Figures S1A, B**). We also measured feeding rates in *Dicer-2* and *YW* following 1 hour starvation to reduce possible irregular feeding patterns, which showed similar results to non-starved flies (**Figures S2A, B**). Collectively, these results show that ZIKV infection alters *Drosophila* feeding rates in a sex-dependent manner, suggesting severe motor and metabolic dysfunction as disease outcomes.

### ZIKV-Infected Flies Show Perturbed Carbohydrate Metabolism

**Figure 2:**
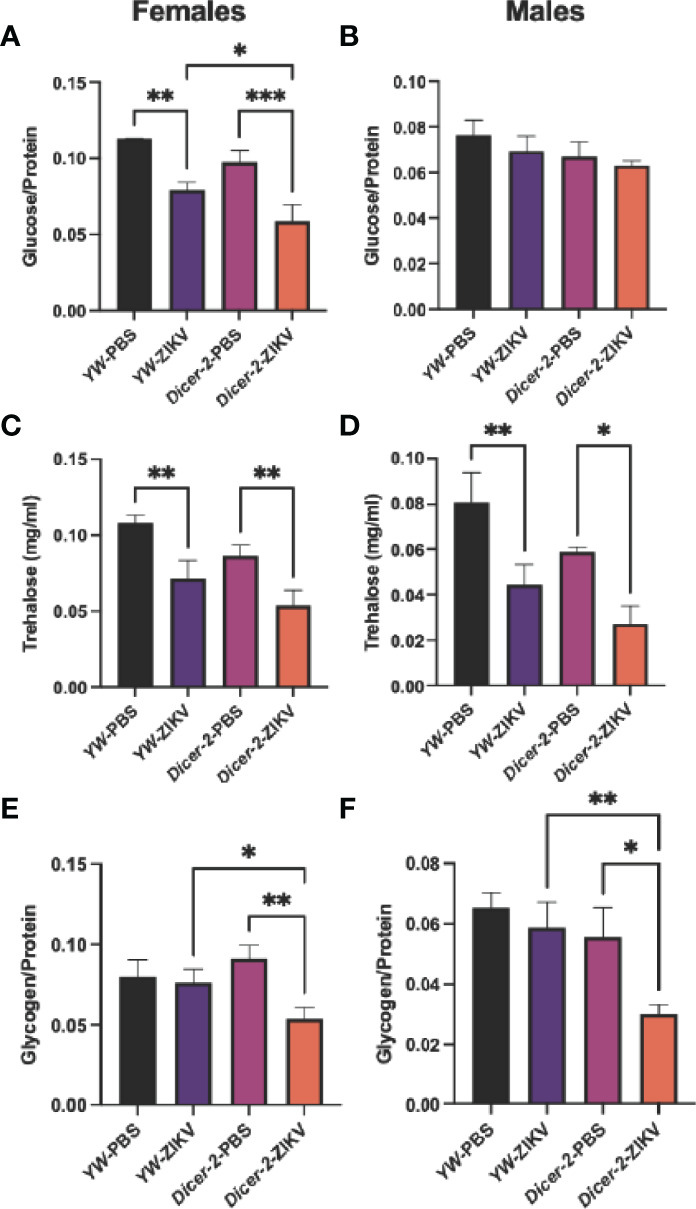
Carbohydrate metabolism in *Drosophila melanogaster* 4 days after infection with Zika virus (ZIKV). **(A, B)** Glucose levels in infected female and male *Dicer-2* mutant adults compared to *YW* (background line) and PBS (no infection treatment) controls. **(C, D)** Trehalose levels in ZIKV-infected and PBS-injected *Dicer-2* mutants and *YW* flies. **(E, F)** Glycogen levels in ZIKV-infected and PBS-injected *Dicer-2* mutants and their *YW* background controls. All experiments were performed in duplicates and repeated three times. (One-way ANOVA, *p < 0.01, **p < 0.001, ***p < 0.0001).

To further characterize the ZIKV-induced pathology, we next examined the effect of ZIKV infection on host metabolism, which is essential for cellular energy maintenance and survival during infection (27). *Drosophila* regulates glucose and trehalose (a disaccharide of glucose) as circulating carbohydrates, and stores excesses in the form of glycogen and lipids in the fat body (35). We first assessed all three types of *Drosophila* carbohydrates in *Dicer-2* mutants and *YW* adult flies at 4 days following injection with the MR766 strain. Both glucose and trehalose levels were strongly reduced in ZIKV-infected females compared to their respective PBS-treated controls (**Figures 2A, C**). More specifically, ZIKV-infected, *Dicer-2* females had lower levels of glucose in comparison to ZIKV-infected *YW* flies, indicating a positive correlation between the RNAi pathway and metabolism (**Figure 2A**). We found no significant differences in glucose levels in male flies, regardless of their experimental conditions (**Figure 2B**). However, trehalose levels in infected *Dicer-2* and *YW* flies were lower in comparison to their PBS-treated controls (**Figure 2D**).

Glycogen levels in *Dicer-2* female mutants infected with ZIKV also decreased compared to their infected *YW* backgrounds and PBS treatment controls (**Figure 2E**). Interestingly, despite the lack of change in glucose levels in *Dicer-2* male mutants and their controls, glycogen from the same treatment groups showed similar results to female *Dicer-2* mutants, in which glycogen levels in infected *Dicer-2* mutants were lower compared to their infected *YW* backgrounds and PBS treatment controls (**Figure 2F**). Together, these results assert the sex-dependent ZIKV pathology while suggesting a key role for carbohydrate metabolism during ZIKV infection.

**Figure 3:**
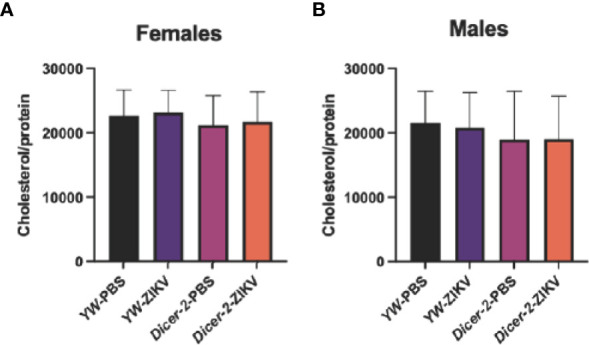
Cholesterol levels in *Drosophila melanogaster Dicer-2* mutant adult flies and their *YW* background controls infected with Zika virus (ZIKV) or injected with PBS (uninfected controls). Quantification experiments were performed in biological duplicates and repeated three times using female **(A)** and male **(B)** individuals. (One-way ANOVA).

Because lipids form the primary source of energy reserve, we also explored whether ZIKV infection triggers cholesterol changes in *Drosophila* hosts. Unlike the observed changes in carbohydrate metabolism, we found no differences among female and male groups (**Figures 3A, B**).

### ZIKV Infection Suppresses the Insulin Pathway in *Dicer-2 Drosophila* Mutants

**Figure 4:**
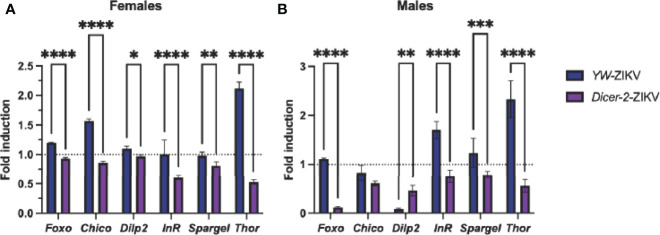
Analysis of insulin-regulated gene expression through quantitative RT-PCR in Zika virus (ZIKV)-infected *Drosophila melanogaster Dicer-2* mutants and *YW* background control flies compared to uninfected (PBS-injected) background controls. Levels of mRNA were normalized against *RpL32* (housekeeping gene), and three independent experiments were performed using adult females **(A)** and males **(B)**. (Two-way ANOVA, *p = 0.0252, **p = 0.0018, ***p = 0.0009, ****p < 0.0001).

The canonical RNAi pathway is closely associated with the insulin signaling pathway, as both have been established as key determinants in vector competence and disease outcome (7, 36). Studies using *Drosophila* demonstrate that insulin signaling presumes an antiviral role by promoting the expression of the RNAi components *Dicer-2* and *Ago-2* (36, 37). Thus, we asked whether ZIKV infection affects insulin gene expression in *Dicer-2* mutants and *YW* adult flies. We found a strong downregulation of insulin-regulated genes *foxo, chico, dilp2, InR, spargel*, and *Thor* in infected *Dicer-2* female mutants compared to their infected *YW* background controls (**Figure 4A**). Similar to these findings, *Dicer-2* male mutants showed low expression levels in all insulin genes except for *chico* (**Figure 4B**). Collectively, this indicates a significant correlation between the RNAi and insulin pathways in *Drosophila* in the context of ZIKV infection.

### Insulin Signaling Promotes *Drosophila* Resistance Against ZIKV Infection

**Figure 5:**
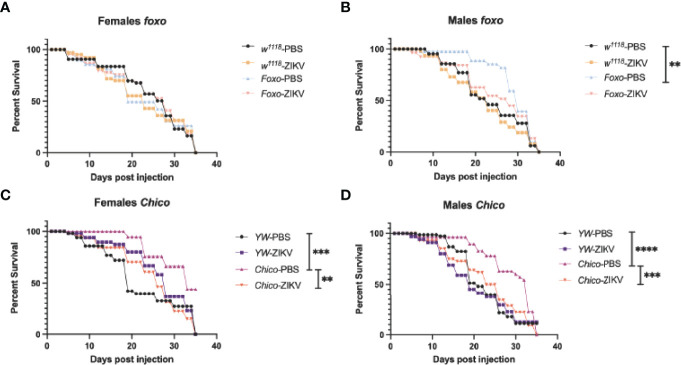
Survival of *Drosophila melanogaster* adult flies after intrathoracic injection with Zika virus (ZIKV) was monitored at 24-h intervals for 35 days. Injections with PBS served as negative controls. **(A, B)** Survival of *FOXO* female and male mutants compared to their background (*w^1118^*) controls. **(C, D)** Survival of *Chico* female and male mutants compared to *YW* background controls. Data represent three biological replicates of at least 20 adult flies. Log-rank (Mantel–Cox) was used for statistical analysis, ^**^p = 0.0033, ^***^p = 0.0001, ^****^p < 0.0001.

**Figure 6:**
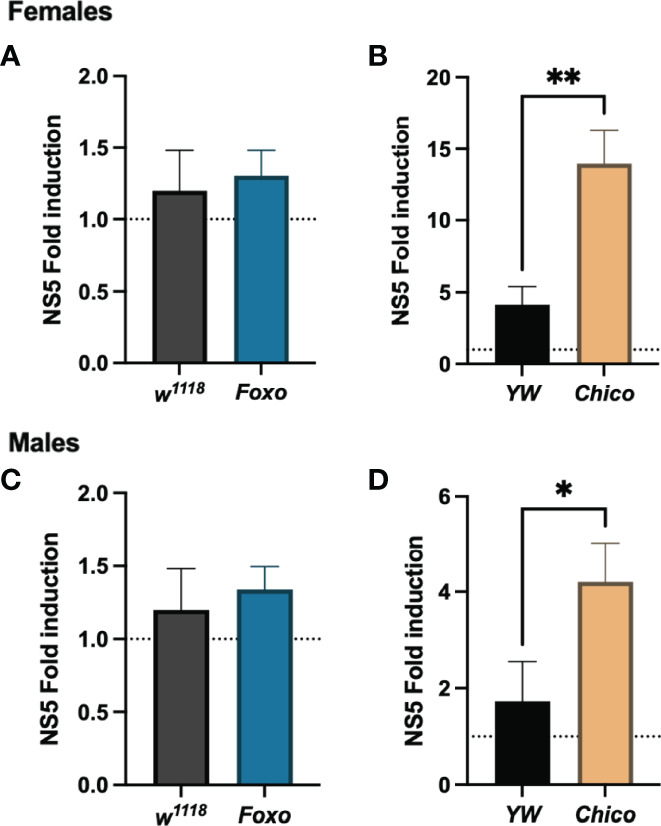
Zika virus (ZIKV) load using qRT-PCR analysis and *NS5* gene-specific primers in *Drosophila melanogaster Foxo* and *Chico* mutants compared to their background controls *YW* and *w^1118^*, respectively. Data, collected from female **(A, B)** and male **(C, D)** adult flies, were normalized to the housekeeping gene *RpL32* shown relative to control flies injected with PBS (injury control). Three independent experiments were carried out with 10 flies per sample in technical duplicates (One-way ANOVA, *p < 0.01, **p < 0.001).

To understand whether insulin signaling modulates survival to ZIKV infection in *Drosophila*, we injected flies carrying loss-of-function mutations in *foxo* and *chico* with MR766 and estimated survival rates over time. We found that infection with this ZIKV strain failed to reduce fly survival in both female and male *foxo* mutants, which were similar to the survival of PBS-injected controls and background flies (**Figures 5A, B**). In contrast, female and male *chico* mutants succumbed to ZIKV infection at a much faster rate compared to their background controls, indicating an essential role for *chico* in host immunity against ZIKV (**Figures 5C, D**). We then estimated ZIKV copy numbers in the infected flies compared to PBS control-treated flies at 4 days post injection using primer sequences against NS5, the largest and most critical nonstructural protein in the ZIKV replication complex (38–40). In corroboration with the previous findings, we found strongly elevated levels of ZIKV copies in both female and male *chico* mutants compared to their respective controls (**Figures 6B, D**). *foxo* mutants on the other hand showed no difference in fold induction compared to their controls (**Figures 6A, C**), which further emphasizes that *chico* plays an antiviral role in *Drosophila* during ZIKV infection.

### Insulin Promotes RNAi Signaling to Fight ZIKV Infection in *Drosophila*

**Figure 7:**
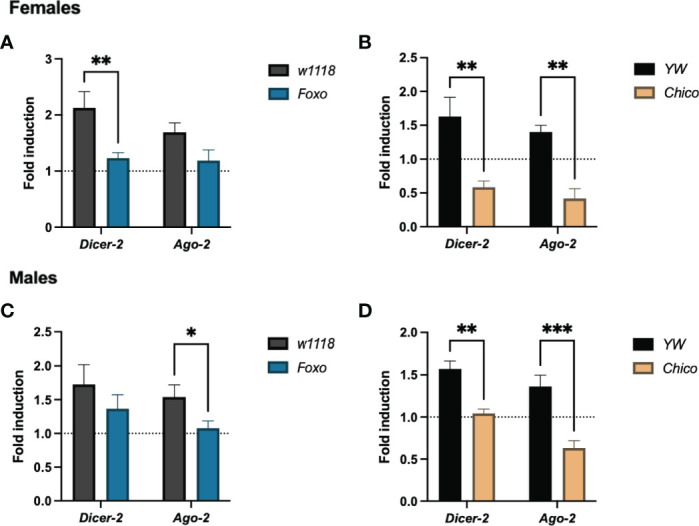
RNAi signaling activity in Zika virus (ZIKV)-infected *Drosophila melanogaster* insulin mutants. Mutant adult flies for *Foxo* and *Chico* were processed for RNA analysis and gene expression levels were determined by qRT-PCR at 4 days post infection. Transcript levels of the RNAi genes *Dicer-2* and *Ago-2* in females **(A, B)** and males **(C, D)** are shown (Two-way ANOVA, *p = 0.0272, **p < 0.001, ***p = 0.0005).

To test whether insulin signaling confers RNAi resistance to ZIKV infection in *Drosophila* hosts, we estimated the transcript levels of *Dicer-2* and *Ago-2* in *foxo* and *chico* null mutants. *Dicer-2* was significantly reduced in infected *foxo* and *chico* female mutants (**Figures 7A, B**), whereas *Ago-2* decreased only in infected *chico* female mutants (**Figure 7A**). On the other hand, *Dicer-2* expression levels were strongly reduced in *chico* male mutants only, while *Ago-2* levels were lower in both in *foxo* and *chico* males (**Figures 7C, D**). This indicates that insulin potentiates RNAi signaling in *Drosophila* and renders resistance to ZIKV infection differently in both sexes.

## Discussion

*Drosophila* possesses distinct antiviral immune mechanisms that are found in both mosquitos and vertebrate animals, thus providing a suitable model for studying host-arbovirus interactions (6–10, 41). Our previous work showed that *Drosophila* flies can sustain the replication of the ZIKV African strain MR766 following intrathoracic injection of the virus (11). This work also highlighted the RNAi component *Dicer-2* as a key regulator of fly immunity against ZIKV, which is closely associated with host homeostasis and metabolic processes. Using *Dicer-2* mutant flies, here we show that ZIKV infection results in behavioral and metabolic shifts that promote a successful replication of the virus and progression of the disease, therefore uncovering viral mechanisms aimed to manipulate host immune and metabolic systems. Our findings show that ZIKV infected *Dicer-2* female mutants display strikingly reduced food consumption rates compared to *YW* background and uninfected controls, which signifies neurobehavioral modifications triggered by the virus. Interestingly, infected *Dicer-2* male mutants do not exhibit the same behavior, indicating sex-dependent deficits caused by ZIKV infection. Similar differences have been reported in mouse models, in which sex-dependent, long-term behavioral abnormalities after ZIKV exposure were observed (42). These findings establish a foundation for identifying susceptibility factors that lead to the development of ZIKV pathologies and secondary behavioral changes in infected individuals.

Although many viruses enhance their replication by reprogramming host metabolism, whether ZIKV drives similar modifications, and the functional consequences of Zika-induced metabolic changes, remain largely unclear. Our findings show that ZIKV infection alters carbohydrate metabolism in *Dicer-2* female mutants by targeting glucose, trehalose, and glycogen levels, which are critical regulators of the glycolysis pathway in *Drosophila* (43, 44). Infected *Dicer-2* male flies also show a significant decrease in glycogen levels despite the lack of change in the other carbohydrates. Noticeably, Dengue virus (DENV), which is closely related to ZIKV, depends on the glycolysis pathway for its replication (45), and high glucose levels have been shown to restrict ZIKV infection in human kidney cells (46). In line with our findings, recent studies in human cells also report dramatic changes in carbohydrate concentrations after ZIKV infection (47). These alterations target proteins including enzymes, transcription regulators, transporters, and kinases associated with energy generation and immune cell activation. Thus, the downregulation of glucose, trehalose, and glycogen suggests that ZIKV hijacks host carbohydrate metabolism to secure the energy required for replication and virion production (48). Consequently, the glycolysis pathway could be a potential target against ZIKV and needs further investigation in the future.

It was previously also shown that ZIKV modulates host cell lipid metabolism, resulting in reduced fat body lipid droplets in *Dicer-2* female mutants (11). More precisely, the reduced size of lipid droplets was linked to the decrease of triacylglycerides stores, causing lipodystrophy, a severe effect of ZIKV infection on fat body homeostasis (49). Our results show no cholesterol level differences among the various *Drosophila* experimental groups, suggesting that cholesterol is not involved in fly resistance against ZIKV and confirming triacylglycerols as the primary candidates for additional studies on lipid metabolism and energy homeostasis in the context of ZIKV infection.

The reduced metabolite levels can be also attributed to insulin signaling, which regulates both carbohydrate and lipid metabolism to maintain nutritional homeostasis and energy reserves during infection (27, 43, 49). In fact, *Drosophila* insulin signaling has been implicated in host antiviral defense by activating several other canonical immune pathways including RNAi, JAK/STAT, and autophagy, all of which are key determinants in vector competency and disease outcome (7, 11, 26, 36). Our results present evidence of severe reduction in insulin gene expression in both *Dicer-2* female and male mutants, therefore revealing insulin signaling as a target involved in the regulation of ZIKV infection. To determine the correlation between the RNAi and insulin pathways during ZIKV infection, we used gene-specific primers to estimate *Dicer-2* and *Ago-2* expression in insulin mutants. It should be of note that gene expression was estimated between fly lines of the same background in this study to ensure similar basal levels of *Dicer-2* and *Ago-2* before viral infection (**Figures S3A–D**). Interestingly, infected female *foxo* and *chico* mutants exhibited a downregulation of *Dicer-2*, but only *chico* loss-of-function female mutants had a strong decrease in *Ago-2*. In contrast, infected male *foxo* and *chico* mutants presented reduced expression of *Ago-2*, but only *chico* male mutants had a reduced *Dicer-2* expression. These sex-specific differences, parallel to the behavioral changes observed earlier, call for future exploration and explicit reporting of mechanisms underpinning immunity in both sexes. Overall, these results indicate a strong link between RNAi and insulin signaling, through which *Drosophila* hosts can combat ZIKV infection.

To further characterize the antiviral role of insulin signaling during ZIKV infection, we show for the first time that *chico*, which encodes an insulin receptor substrate that functions in the *Drosophila* insulin/insulin-like growth factor (IGF) signaling pathway, is required for an insulin-mediated antiviral response. *Chico* regulates the *Drosophila* insulin receptor (InR), which controls a diverse array of biological processes including cellular metabolism (50). Its essential role in cell growth regulation is confirmed by the small size of *chico* mutants, which are half the size of normal flies (51). Most importantly, the similarities of growth defects caused by *chico* mutations in *Drosophila* and insulin/IGF1 signaling pathway in vertebrates suggest that this pathway, *chico* in particular, plays a conserved role in antiviral immunity and metabolism during ZIKV infection (52, 53).

Remarkably, loss of function in the transcription factor *foxo* does not compromise fly survival ability in either female or male experimental groups upon ZIKV infection. *Foxo* is known for its association with nutritional signaling and induction of RNAi components during arboviral infection (54). However, the complex nature and interplay between immune pathways complicates endeavors to characterize the distinct roles of insulin signaling components, making imperative to further investigate *foxo* gene expression and the specific mechanism that insulin contributes to antiviral immunity. Higher viral copy numbers in the host also corresponded to the survival trends observed in this study. Infected *chico* mutants, both females and males, showed significantly increased levels of NS5 compared to background and PBS controls-treated flies. Together, these results confirm *chico* as indispensable for fly survival and resistance against ZIKV infection.

Taken together, our results show that *Drosophila* provides an ideal model system for identifying key regulators of host metabolism and revealing their vital functions in homeostasis and immunity during viral infection. Our findings reveal that ZIKV dramatically modifies host metabolism to ensure an optimal environment for its replication and spread. A better understanding of these metabolic alterations required for arboviruses, such as Zika, could lead to novel therapeutic approaches through targeted inhibition of specific cellular metabolic pathways.

## Data Availability Statement

The raw data supporting the conclusions of this article will be made available by the authors, without undue reservation.

## Author Contributions

GT-E designed and conducted the experiments, analyzed the data, constructed the figures, interpreted the results, and wrote drafts of the manuscript. AK conducted the experiments, analyzed the data, and constructed the figures. IE designed the experiments, interpreted the results, and revised the manuscript. All authors contributed to the article and approved the submitted version.

## Funding

This work was supported by a supplement to grant R01AI110675 to IE from the National Institute of Allergy and Infectious Diseases.

## Conflict of Interest

The authors declare that the research was conducted in the absence of any commercial or financial relationships that could be construed as a potential conflict of interest.

## Publisher’s Note

All claims expressed in this article are solely those of the authors and do not necessarily represent those of their affiliated organizations, or those of the publisher, the editors and the reviewers. Any product that may be evaluated in this article, or claim that may be made by its manufacturer, is not guaranteed or endorsed by the publisher.
