# Supplementary material for: Zika Virus Induces Sex-Dependent Metabolic Changes in Drosophila melanogaster to Promote Viral Replication
Source: Front Immunol. 2022 Jun 30;13:903860. doi: 10.3389/fimmu.2022.903860 (PMC9280044; doi:10.3389/fimmu.2022.903860)
Supplement: Supplementary file 1 [file DataSheet_1.docx]

**
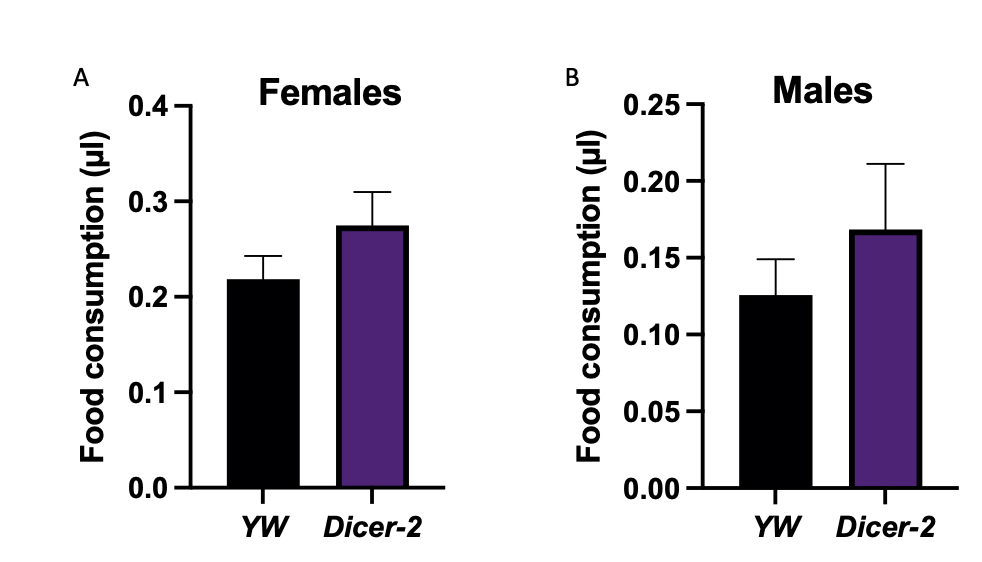
**

**Figure S1**. Feeding rates of untreated *Drosophila melanogaster* adult flies. **(A)** Untreated *Drosophila* female adult *Dicer-2* mutants and their background control (*YW*). **(B)** Untreated *Drosophila* male adult *Dicer-2* mutants and their background control (*YW*).


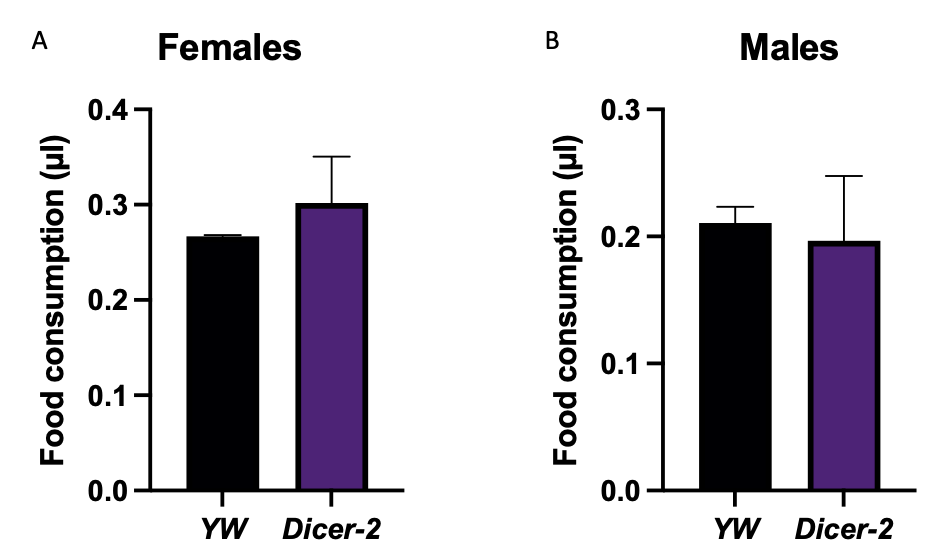


**Figure S2**. Feeding rates of untreated *Drosophila melanogaster* female adult *Dicer-2* mutants **(A)** and male adult *Dicer-2* mutants **(B)** and their respective background controls (*YW*) following 1 hour starvation.


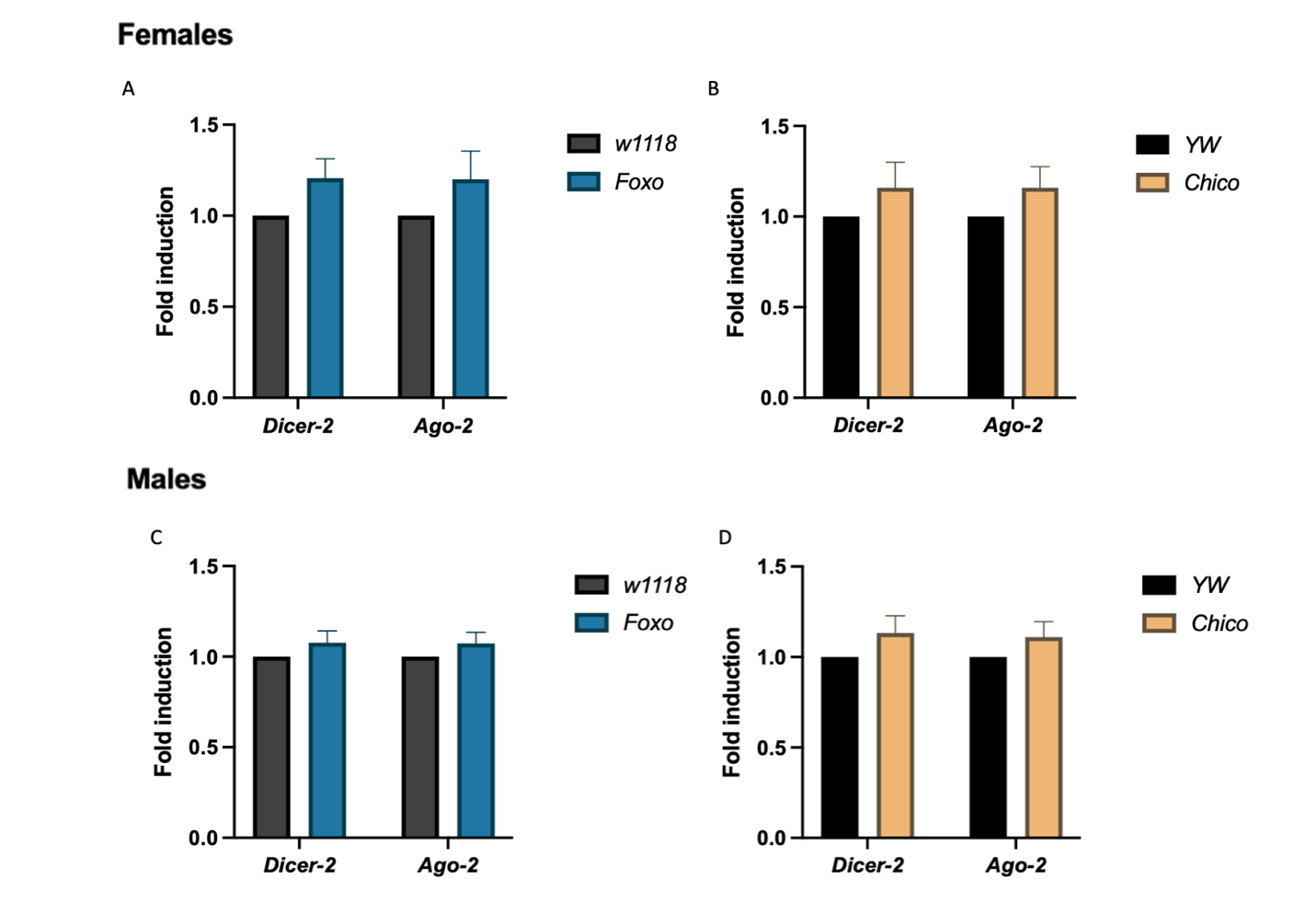


**Figure S3**. Basal RNAi signaling activity (*Dicer-2* and *Ago-2* gene expression) in untreated *Drosophila melanogaster* female and male adult *dFOXO* **(A, C)** and *chico* **(B, D)** mutant flies.
